# Comparison of pharmacy-based measures of medication adherence

**DOI:** 10.1186/1472-6963-12-155

**Published:** 2012-06-12

**Authors:** William M Vollmer, Maochao Xu, Adrianne Feldstein, David Smith, Amy Waterbury, Cynthia Rand

**Affiliations:** 1 Kaiser Permanente Center for Health Research, Portland, OR, USA; 2 Illinois State University, Normal, IL, USA; 3 Johns Hopkins University, Baltimore, MD, USA; 4 Center for Health Research, 3800 N Interstate Avenue, Portland, OR, 97227-1110, USA

**Keywords:** Asthma, Inhaled corticosteroids, Adult, Medication adherence, Randomized clinical trial, Methodology

## Abstract

****Background**:**

Pharmacy databases are commonly used to assess medication usage, and a number of measures have been developed to measure patients’ adherence to medication. An extensive literature now supports these measures, although few studies have systematically compared the properties of different adherence measures.

****Methods**:**

As part of an 18-month randomized clinical trial to assess the impact of automated telephone reminders on adherence to inhaled corticosteroids (ICS) among 6903 adult members of a managed care organization, we computed eight pharmacy-based measures of ICS adherence using outpatient pharmacy dispensing records obtained from the health plan’s electronic medical record. We used simple descriptive statistics to compare the relative performance characteristics of these measures.

****Results**:**

Comparative analysis found a relative upward bias in adherence estimates for those measures that require at least one dispensing event to be calculated. Measurement strategies that require a second dispensing event evidence even greater upward bias. These biases are greatest with shorter observation times. Furthermore, requiring a dispensing to be calculated meant that these measures could not be defined for large numbers of individuals (17-32 % of participants in this study). Measurement strategies that do not require a dispensing event to be calculated appear least vulnerable to these biases and can be calculated for everyone. However they do require additional assumptions and data (e.g., pre-intervention dispensing data) to support their validity.

****Conclusions**:**

Many adherence measures require one, or sometimes two, dispensings in order to be defined. Since such measures assume all dispensed medication is used as directed, they have a built in upward bias that is especially pronounced when they are calculated over relatively short timeframes (< 9 months). Less biased measurement strategies that do not require a dispensing event are available, but require additional data to support their validity.

****Trial registration**:**

The study was funded by grant R01HL83433 from the National Heart, Lung and Blood Institute (NHLBI) and is filed as study NCT00414817 in the clinicaltrials.gov database.

## **Background**

Pharmacy databases are increasingly being used to assess medication usage, and a number of measures have been developed to characterize both the intensity of medication usage compared to intended use (adherence), and the extent to which such usage persists over time (persistence) [1,2]. Such measures have strong appeal, and an extensive literature now exists to support their value in clinical research [3]. In particular, comparative effectiveness research increasingly depends on pharmacy-based measures to assess both practice variations in prescribing, as well as health outcomes according to therapeutic exposure in real-world, diverse patient populations. Despite their increasingly widespread use, however, relatively few studies have systematically compared the properties of competing pharmacy-based measures of adherence [1,4-6].

One of the most widely cited pharmacy-based measures of medication adherence is calculated simply as the total number of days of medication dispensed between some initial and final dispensing date divided by the length of time between these dates. Steiner and Prochazka [7] refer to this and related indices as part of a larger class of measures they call continuous multiple-interval measures of medication availability (or CMA measures for short). They also define a related class of measures they term continuous multiple-interval measures of medication gaps (or CMG measures) that attempt to quantify theoretical gaps in medication usage. CMA-based measures are easier to compute than CMG-based measures and make no assumptions about patterns of medication use, whereas CMG measures are computationally more intensive and assume that medications, once dispensed, are used as directed.

Both CMA-based and CMG-based measures were originally developed under the assumption that one has access only to dispensing data during some defined observation window and knows nothing else about the individuals under observation. Possible refinements include (1) expanding the denominator to include the entire observation window under study if it is known that the individuals under study should be taking a given medication (e.g., by clinical practice guideline recommendation) throughout the observation window and (2) accounting for medications that individuals are known to possess at the start of the observation window. The proliferation of electronic medical records (EMRs) is making access to such information increasingly more common.

We used data from an adherence trial among members of a large health maintenance organization to compare the properties and performance characteristics of a variety of CMA- and CMG-based measures of medication adherence.

## **Methods**

### **Study design and research setting**

We report data collected as part of a randomized clinical trial of members of the Northwest (KPNW) and Hawai‘i (KPH) regions of Kaiser Permanente, a large, group-model health maintenance organization. The main results of the study have been presented elsewhere [8]. Participants were randomized to receive either usual care or an 18-month intervention in which automated telephone calls were used to promote improved adherence to inhaled corticosteroids (ICS). The study was approved by the Institutional Review Boards (IRBs) of each region (Kaiser Permanente Northwest IRB, FWA #00002344-00000405; and Kaiser Permanente Hawaii IRB, FWA #00002344-00000402) and written informed consent was waived. Research was carried out in compliance with the Helsinki Declaration.

Kaiser Permanente provides comprehensive, prepaid health care services to its members, and both KPNW and KPH utilize a fully electronic medical record. An end-user database captures diagnostic-specific utilization data, procedures performed, new medication orders and outpatient pharmacy dispensings.

### **Study population**

The target population for the trial consisted of KPNW and KPH members aged 18 and older who were members for the 12 months prior to randomization, had been seen for asthma and received at least one dispensing of a respiratory medication during that timeframe. In order to be able to study both primary and secondary ICS adherence, the target population included individuals without evidence of prior ICS use. The present analysis focuses on the subset of 6903 individuals with ICS dispensings during the baseline year.

### **Adherence measures**

We compare eight alternative measures of adherence, which we label CMA1-CMA8 (Table 1). Some of these are classical CMA-type measures and some are derived from CMG-like measures. In each case we assume an *observation window*, defined as the period from randomization (t_0_) to end of follow-up (t_e_). We also treat all ICS medications, including combination agents containing an ICS, as a single class of medications.

**Table 1:** Summary of study measures

**Defn**	**start of window to first dispensing**	**last dispensing to end of window**	**Timing**^**^**^	**Description of Measure**
**CMA1**	ignored	ignored	ignored	(# days dispensed, excluding last) / (first to last dispensing)
**CMA2**	ignored	counted	ignored	(# days dispensed, including last) / (first dispensing to end of window)
**CMA3**	ignored	ignored	ignored	minimum (CMA1, 1)
**CMA4**	ignored	counted	ignored	minimum (CMA2, 1)
**CMA5**	ignored	ignored	counted	(# days theoretical use^#^) / (first to last dispensing)
**CMA6**	ignored	counted	counted	(# days theoretical use^#^) / (first dispensing to end of window)
**CMA7**	counted	counted	counted	(# days theoretical use^#^) / (start to end of observation window), includes in numerator meds carried into observation window
**CMA8**	counted	counted	counted	(# days theoretical use^#^) / (lagged* start of obs’n window to end of window), numerator and denominator ignore intial lag period

^^^refers to whether the timing of the dispensing (actual date dispensed) is taken into account in the calculations or is ignored.

^#^assumes medications taken as directed and new medications “banked” until needed.

*lag refers to initial period covered by medication supply on hand at start of observation window. Medications dispensed during lag interval are “banked” and counted starting with end of lag.

For any given participant, let t_1_, t_2_, …, t_k_ be the dates of the *k* ICS dispensing events that occurred during the observation window, and n_1_, n_2_, …, n_k_ be the corresponding number of days of medication dispensed at each timepoint. For any given dispensing event, we calculated days' supply by dividing the number of puffs per canister by the doctor’s suggested daily usage instructions. Using this information, we define CMA1 as the total days’ supply of ICS dispensed between the first and last dispensing event, excluding medications dispensed at the final dispensing event, divided by the elapsed time between these events. That is, CMA1 = (n_1_+ n_2_+ … + n_k-1_) / (t_k_ – t_1_).

We shall refer to the denominator in these formulas, in this case t_k_ – t_1_, as the *measurement window* (i.e., the subset of the larger observation window over which adherence is actually calculated). Implicit in the use of the above definition is the assumption that we don’t know whether the participant should be taking the medication of interest until we see a dispensing for it. That is why the denominator starts with t_1_ rather than t_0_. Similarly the dispensing at t_k_ implies that the participant was still taking the medication at time t_k_. However since we don’t know if use of the medication was subsequently discontinued under physician directions, observation stops at t_k_ rather than at t_e_. Thus two dispensing events are required to calculate the measure.

The first refinement to CMA1 tacitly assumes the medication should continue to be taken during the interval from t_k_ to t_e_ (a period sometimes referred to as the “terminal gap”) [9]. It is computed as the total days’ supply dispensed during the observation window, including the medication dispensed at t_k_, divided by the time from t_1_ to end of study: CMA2 = (n_1_+ n_2_+ … + n_k_) / (t_e_ – t_1_).

In contrast to CMA1, CMA2 requires only one dispensing event to be calculated. In the case of asthma, a chronic condition for which ICS is considered first line therapy, this assumption would seem to be reasonable, especially if the observation window does not extend for several years past t_k_. Because CMA1 and CMA2 may both be greater than 1, some researchers prefer to cap them at 1 (since nominally adherence shouldn’t be any greater than 100 %). This gives rise to two additional measures, CMA3 = minimum (CMA1, 1) and CMA4 = minimum (CMA2, 1).

The terminology used to describe these measures in the literature is not consistent. Hess et al. [1] refer to CMA1 as the *Compliance Rate* and CMA2 as the *Continuous Measure of Medication Acquisition*. CMA3 and CMA4 appear to be examples of what Hess et al. refer to as the *Proportion of Days Covered*, although it is not clear from their description of this measure exactly what denominator they use in this calculation, only that it is capped at 1. In practice many authors also refer to both CMA1 and CMA2 as the *Medication Possession Ratio (MPR)*, and indeed Hess et al. [1] note that at least four different published measures have been ascribed this name. By contrast they seem to use MPR (and equivalently the *Medication Refill Adherence* percentage) to refer to the total days’ supply divided by the entire observation window [i.e., (n_1_+ n_2_+ … + n_k_) / (t_e_ – t_0_)].

The preceding are all classical CMA-type measures in that they do not account for the timing of the dispensing events. An individual can have long gaps with no medication dispensings and yet appear fully compliant based on a large days’ supply dispensed toward the end of the measurement window. The CMG-based measures were developed to address this potential limitation. If one assumes that all medication is taken exactly as directed, then it is possible to determine those days for which an individual is theoretically taking medication and conversely those days for which no medication is available and hence gaps in coverage exist. For instance, if a participant receives a 30-day supply of ICS on day 10 of an observation window and another 30-day supply on day 50, then he must have had a 10-day coverage gap from days 40–49 if the medication was taken as directed. In the event of overlapping dispensings, it is conventional to assume that the new dispensing is not started until the first is exhausted. Thus in the preceding example if the second dispensing occurred on day 35, it would be “banked” and not started until day 40. Traditionally, CMG measures are used to reflect the proportion of measurement window in which medication is not taken and the duration of usage gaps, but equivalently one can use 1-CMG to estimate the proportion of days during a measurement window during which a participant was taking medication. Similar to the distinction between CMA1 and CMA2, we can therefore define CMA5 = (# adherent days between t_1_ and t_k_) / (t_k_-t_1_) and CMA6 = (# adherent days between t_1_ and t_e_) / (t_e_-t_1_).

That is, CMA5 ignores the last dispensing while CMA6 does not. In both cases any unused medication still available at the end of the observation window is ignored in the calculations. Thus both measures are by definition bounded above by 1 and hence along with CMA3 and CMA4 are variants of Hess et al.’s [1]*Proportion of Days Covered* measure, and also closely aligned with what they term the *Continuous Measure Of Medication Gaps*.

The definitions of CMA1-CMA6 are predicated on the assumption that no other information is known about the study participants. In particular it is unknown whether they should be taking ICS at the start of the observation window (and potentially if they are bringing any unused medication into the start of the observation window). This need not be the case. For instance, in our study we had access to data from the EMR for the “baseline” year prior to randomization. Given that we knew each participant had a diagnosis of asthma and it was severe enough to have been prescribed an ICS in the baseline year, we felt it reasonable to assume that the participant should be taking an ICS throughout the entire 18-month follow-up window. Indeed the intervention calls were intended in part to help restart ICS use in those participants who had discontinued their use. We therefore considered two additional CMG-based measures.

CMA7 extends CMA6 by measuring adherence from t_0_ rather than from t_1_ (that is, the measurement window coincides with the observation window from t_0_ to t_e_). In addition, we adjusted the numerator to include any medication still available based on the most recent dispensing prior to randomization. Thus CMA7 = (# adherent days between t_0_ and t_e_) / (t_e_-t_0_). (Given the assumptions we made about ICS use, it didn’t make sense to consider an analog to CMA5 that only spanned the interval t_0_ to t_k_.) Finally, since the initial adherence resulting from medications already on hand at the start of the intervention could not have been influenced by our intervention, we considered one further refinement to this definition, which was to start the observation window on the day this initial prescription should have been exhausted. If we label this date as t_0_*, we have CMA8 = (# adherent days between t_0_* and t_e_) / (t_e_-t_0_*).

The initial days’ supply on hand at the time of randomization is not counted in the numerator and the corresponding amount of time is also subtracted from the denominator. An important feature of CMA7 and CMA8 is that they do not require a dispensing during the observation window to be calculated and hence can be defined for all participants.

### **Statistical methods**

We use simple descriptive statistics to compare the performance characteristics of these eight measures. For some analyses we also calculate CMA measures, and their associated statistics, for varying lengths of follow-up (e.g., the first 3, 6, and 9 months of follow-up). We limited these latter analyses to those individuals with complete follow-up through 15 months (n = 4790, ≈69 % of the full cohort) to assure that any differences were not attributable to patient mix. All statistical analyses were performed using SAS v9.1 (SAS Institute, Cary, NC).

## **Results**

Summary descriptive statistics for the various adherence measures are displayed in Table 2. As shown, the number of individuals for whom we can compute valid values varies directly as a function of how these terms are defined. CMA1, CMA3, and CMA5 all require the presence of two or more dispensings to be calculated, and hence could be defined for only 68 % of the population. CMA2, CMA4, and CMA6 require only a single dispensing to trigger the calculation, and for our data could be calculated for 83 % of the population. Finally, CMA7 and CMA8 are available for everyone since they require no dispensings to calculate.

**Table 2:** Comparison of distributional characteristics of competing medication acquisition measures

	**CMA1**	**CMA2**	**CMA3**	**CMA4**	**CMA5**	**CMA6**	**CMA7**	**CMA8**
N	4661	5716	4661	5716	4661	5716	6903	6903
Mean	0.61	0.63	0.55	0.52	0.54	0.50	0.36	0.35
Std Dev	0.94	1.52	0.29	0.31	0.29	0.30	0.30	0.30
Percentiles								
min	0.02	0.01	0.02	0.01	0.02	0.01	0.00	0.00
1^st^	0.07	0.05	0.07	0.05	0.07	0.05	0.00	0.00
5^th^	0.12	0.09	0.12	0.09	0.12	0.09	0.00	0.00
25^th^	0.29	0.25	0.29	0.25	0.29	0.24	0.10	0.09
50^th^	0.51	0.48	0.51	0.48	0.51	0.45	0.29	0.27
75^th^	0.80	0.80	0.80	0.80	0.79	0.74	0.58	0.57
95^th^	1.24	1.33	1.00	1.00	1.00	1.00	0.95	0.95
99^th^	1.94	2.54	1.00	1.00	1.00	1.00	1.00	1.00
max	50.00	100.00	1.00	1.00	1.00	1.00	1.00	1.00

Medication acquisition reported as a fractional measure, e.g., 0.61 = 61 %. Percentiles illustrate the medication acquisition distribution in each measure’s population, e.g., the median for CMA1 = 0.51.

Table 2 also shows the relative upward bias in CMA1-CMA6 (and especially CMA1 and CMA2) compared to CMA7 and CMA8. Because CMA1 – CMA6 all require at least one dispensing to be calculated, they by definition guarantee some level of adherence. This is not the case for CMA7 and CMA8. The even more pronounced relative upward bias in CMA1 and CMA2, which is reflected in their means and standard deviations but not their more robust interquartile ranges, results from the extreme skewness in the right-hand tail of these measures.

Although follow-up in these participants ranged from 1 to 18 months, the average duration of follow-up [mean (SD)] was 15.1 (3.9) months. As shown in Table 3, which presents data for only CMA4, CMA6, CMA7 and CMA8, the upward bias associated with CMA1-CMA6 relative to CMA7 and CMA8 is even more pronounced for shorter observation windows, but still persists even when adherence is measured over a full 15 months. For the most part, the more extreme early bias associated with CMA1-CMA6 (or at least CMA4 and CMA6) appears to largely disappear after 9 months. CMA7 and CMA8 do not exhibit any evidence of a time bias, presumably since they don’t require an initial dispensing (and its attendant implied adherence) to trigger the calculation.

**Table 3:** Impact of window length on measurement properties

**Measure of Medication Aquisition**	**Duration of Observation Window**				
	**3 months**	**6 months**	**9 months**	**12 months**	**15 months**
CMA4	0.68 ± 0.30 (6094)	0.58 ± 0.32 (5545)	0.54 ± 0.32 (4915)	0.53 ± 0.31 (4045)	0.54 ± 0.31 (2694)
CMA6	0.65 ± 0.29 (6094)	0.53 ± 0.29 (5545)	0.50 ± 0.29 (4915)	0.50 ± 0.29 (4045)	0.51 ± 0.29 (2694)
CMA7	0.42 ± 0.33 (6436)	0.41 ± 0.30 (6185)	0.41 ± 0.29 (5855)	0.40 ± 0.29 (5355)	0.40 ± 0.29 (4485)
CMA8	0.35 ± 0.35 (6436)	0.38 ± 0.31 (6185)	0.39 ± 0.30 (5855)	0.39 ± 0.29 (5355)	0.39 ± 0.29 (4485)

Data expressed as mean ± SD (sample size in parentheses).

## **Discussion**

While pharmacy-based dispensing records are becoming increasingly popular tools for studying patterns of medication adherence, it is important to understand the potential limitations of the resulting measures. By their very definition, some measures of adherence are more prone to an upward bias, while other potentially more accurate measures may rely on a stronger set of assumptions to be valid. In addition, such pharmacy-based measures of adherence have face validity as measures of actual medication usage only when measured over long periods of time, since in this case high levels of estimated adherence can only be achieved through repeated refills (which tacitly implies ongoing medication usage).

Although our study was conducted in the context of a randomized clinical trial, we believe our findings have applicability to any pharmacoepidemiologic study that might be conducted using electronic dispensing records. One key consideration is that many of the commonly used measures require medication use during the observation window in order to be calculated. Our study suggests that this requirement will clearly bias adherence values upwards, both by excluding the least adherent patients and by building in a minimum level of adherence in those who can be assessed. Increasingly, investigators will have access to EMRs that can be used to better define the population of individuals who are known users of the medication of interest, or at least have been prescribed such medications, thus allowing more accurate assessments of adherence for a target population. Further, EMR data can be used to identify whether such medication use has been discontinued by the patient’s provider. In this context it is therefore important to understand how many individuals are excluded from your adherence estimates by various definitions, and our results show that this number can be quite large. In the context of comparative studies, where nonadherence can be differential across the groups being compared, it is even more important to account for all subjects.

Of the measures we studied, those that required an initial dispensing for their calculation, particularly if not bounded above by 1 (i.e., CMA1-2), were prone to exhibit an upward bias relative to the other measures we considered. The four variants on the *Proportion of Days Covered*, (CMA3-6), generally performed similarly to one another. Adherence estimates based on all six of these measures exhibited an increasingly upward bias with shorter observation windows that did not flatten out until after about 9 months of observation (Table 3). This reflects the fact that the measurement window for all of these measures begins with a dispensing, and hence a certain amount of built-in implied adherence. The fact that the measurement windows for CMA1, CMA3, and CMA5 also all end with a dispensing event leads to a further upward bias, since they effectively require that one is actively using the medication throughout the measurement window (or at least at both the beginning and end of the window). However, use of CMA2, CMA4, and CMA6 carry the implicit assumption that one should be using the medication throughout the entire interval beginning with the last dispensing event and extending through the end of the observation window (i.e., t_k_ to t_e_). If such an assumption is valid, then these measures should yield more unbiased estimates of adherence than those from their CMA1, CMA3, and CMA5 counterparts. They have the further added advantage that they can be computed for more people since they only require a single dispensing event to be defined. This is particularly important since the missingness pattern will likely not be random (that is, nonadherent individuals will be more likely than adherent individuals to have missing data). Thus the upward bias in CMA1, CMA3, and CMA5 results both from a likely overestimation of adherence in those for whom it can be calculated, as well as from the fact that these same individuals are likely to be selectively more adherent than those for whom these indices cannot be calculated.

If viewed merely as measures of medication acquisition, rather than medication taking, some of the problems discussed above become moot and CMA1 or CMA2 may be the preferred measures to use. Indeed there may be some theoretical interest in those with very high values for CMA1 or CMA2. However, values of CMA1 or CMA2 greater than 1 can also substantially skew mean rates of adherence upwards for a population and in many if not most instances may not reflect actual adherence behavior. To the best of our knowledge based on a limited amount of data checking, the majority of these extreme values reflect actual dispensings rather than, for example, administrative errors in the data. Whether they reflect excessive use by one individual, medication sharing, vacation supplies, or medication wastage we don’t know. Presumably these extreme values reflect some combination of all of these possibilities. In the end, however, we believe that most researchers who use these measures think of and talk about them as measures of adherence, and for that reason we would argue that CMA1 and CMA2 should not be used. Nonetheless, further studies are needed to better understand patient factors associated with very high rates of dispensing, and whether such excessive dispensings are associated with adverse health outcomes. Reasons for this apparent over-adherence have been attributed to changes in directions not noted in the pharmacy record, intentional variable dosing, and stockpiling [10].

In practice, at least for our dataset, we found only minimal differences between CMA3/CMA4 and their CMG-based counterparts CMA5/CMA6. Given that the latter are computationally more intensive to calculate, this might argue for the use of the former measures (which are relatively easy to calculate). The main advantage of using the CMG-based measures is their ability to describe gaps in medication use, although this requires the rather strong assumption that the medication is used exactly as directed. Nonetheless we feel that the added benefit of studying gaps in usage conceptually has a lot of appeal and is worth consideration when evaluating the use of either CMA3 or CMA4 versus CMA5 or CMA6.

Each of CMA1 through CMA6 can be calculated solely on the basis of pharmacy dispensing records available during the observation window. The use of CMA7 and CMA8 requires additional knowledge. This could simply be information about medication use prior to the start of the formal observation window of interest or, as in our case, this information supplemented by diagnostic data from an EMR. Due to the growing use of EMRs and the expectation that they will only become more prevalent over time, these requirements should not pose a serious problem to the future use of these measures. However, the availability of such information, while necessary, is not sufficient to justify the use of CMA7 or CMA8. One must further be able to justify the assumption that participants should be taking the medication throughout the observation window. The validity of this assumption is likely to be more true for some medications than for others. In the case of asthma, for instance, ICS are considered first-line therapy for patients with persistent disease. Hence the presumption that a patient, once prescribed them, should continue using them is more likely to be true than not true (although physicians may discontinue use if they are deemed ineffective for a given patient). The presence of stop orders, if available in the EMR, could be used to further refine the calculation of such measures, although our experience is that clinicians are not good about documenting them.

The fact that CMA7 and CMA8 do not require a dispensing during the observation window in order to be calculated should, if the assumptions underlying their use are met, cause them to lead to the most valid measures of adherence. However, given that the assumptions for their use are inevitably not met for some individuals, in practice population-based estimates of adherence based on these measures are probably biased downwards from truth. These observations are consistent with the trends observed in our data, which showed both the lowest estimates of adherence and the least change in estimated adherence with varying length of the observation window. Even if the assumptions underlying CMA7 and CMA8 do lead to a downward bias in the estimates, this needs to be weighed against the fact that these measures can be calculated for everyone. Despite the fact that everyone had a pre-existing diagnosis of asthma and an order or dispensing for ICS in the baseline year, CMA1/3/5 could not be calculated for 32 % of this cohort, and even the less restrictive CMA2/4/6 measures could not be calculated for 17 % of the cohort.

Although we did not evaluate them as part our analyses, under the assumptions for use of CMA7 and CMA8 one could also modify the definitions of CMA4 and CMA6 to include the entire observation window in the denominator. The modified CMA4 index in particular, which would correspond to Hess et al.’s [1] definition of the *Medication Possession Ratio*, could be much more easily computed than any of the CMG-based measures and it is not unreasonable to hypothesize that it might be fairly comparable in its measurement properties to CMA7 and CMA8. It would also by definition be definable for all subjects, and not just those with one or more dispensing events during the observation window.

Our motivation for defining CMA8 is probably unique to the context of randomized clinical trials, where the concept of implied initial adherence at the start of an adherence intervention that could not be related to treatment allocation is a relevant consideration for analysis.

Our results are consistent with, and extend, the results of previous investigations that have compared the performance of competing measures of adherence in the same dataset. Hess et al. [1] compared 11 measures, though per their descriptions of them it is not clear that these were all mathematically distinct measures. They concluded that the equivalent of what we term CMA1-CMA6 provided essentially the same adherence values, although those measures that were capped at a maximum value of 1 (or 100 %), similar to our CMA3-CMA6 measures, produced adherence estimates that were slightly lower than those that were not. Participants in their study had a mean (SD) observation window of 350 (16) days, and they did not report on the impact of using shorter observation windows. A separate review of pharmacy-based measures of medication adherence, however, noted that the assessment of adherence over short intervals is likely to be imprecise and suggested that the observation window should be long enough to span the expected days’ supply from at least three dispensing events [9]. This agrees well with our own observation (Table 3) that the extreme early bias in these measures takes about 9 months to flatten out (the average ICS days’ supply for this population was 2–3 months). Of course adherence often falls naturally over time, particularly among new users [10] and that is another rationale for prolonged observation of refill behavior. Vink et al. [11] compared the equivalents of our CMA3 and CMA7 when assessed over one year and concluded that the latter had a significantly better area under the curve for classifying a measure of adherence based on chart review.

Several reports have noted the lack of standardization in terminology in the published adherence literature, noting both that the same term (e.g., *Medication Possession Ratio*) can mean different things in different papers and conversely that multiple distinct terms have been used to describe the same measure [1,2,7,9]. These reports also note that comparisons with published studies are further complicated by the fact that the precise methodology used for defining a given measure is not always provided. Our approach has been to explicitly define a series of measures and give their rationales and assumptions for valid use, while avoiding assigning a formal nomenclature to them. We have, however, tried to relate our terms to those used by Hess et al. [1].

In the end, the choice of which adherence measure to use for any given study must be based on the richness of data available to the investigator, the chronicity of the disease for which the medication is being used, the availability of other therapeutic options, knowledge about standards of practice, and the question being addressed by the study. No single measure is likely to be optimal for all occasions. Still, given that the assumptions for use of more sophisticated measures, such as CMA7 or CMA8, are met, we believe our findings suggest that these may be more appropriate (i.e., less biased and more universally estimable) alternatives than simpler measures such as the various derivatives of the MPR.

The study has two main limitations. First, none of the measures described here really speak to the issue of primary nonadherence (failure to obtain the first fill of a medication ordered by their clinician), and instead focus on adherence among known medication users. Thus from the perspective of overall population-based measures of adherence to prescribed medications, all of these measures will tend to have a further upward bias. In addition, we made no attempt to adjust for hospitalization days in our adherence estimates. For populations for which this is a meaningful consideration, it is common to remove hospital days from the denominators of many of these statistics. However, for the purpose of comparing relative properties of the various measures, any bias this introduces is likely to be comparable across the measures so that relative comparisons should be largely unaffected.

## **Conclusions**

Estimates of medication adherence can vary markedly among competing measures.

These differences are accentuated when adherence is measured over relatively short timeframes (< 9 months). Many commonly cited measures cannot be calculated for large numbers of individuals because they require one, or in some cases two, dispensings in order to be defined. This leads to falsely optimistic estimates of adherence. Less biased measurement strategies that do not require a dispensing event to be calculated are available, but require additional data to support their validity.

## **Abbreviations**

ICS, inhaled corticosteroids; CMA, continuous multiple-interval measures of medication availability; CMG, continuous multiple-interval measures of medication gaps; CMA1-CMA8, the eight alternative adherence measures evaluated in this paper (see Table 1 for definitions); SD, standard deviation; KPNW, Kaiser Permanente Northwest Region; KPH, Kaiser Permanente Hawaii Region; EMR, electronic medical record; MPR, medication possession ratio.

## **Competing interests**

The authors declare that they have no competing interests.

## **Author’s contributions**

WV directed the overall parent study, conceived the design of this manuscript, and assumed primary responsibility for drafting it. MX participated in the design of this manuscript, conducted the statistical analyses, and contributed actively to the preparation of the manuscript. AF, DS, AW, and CR all participated in the design and conduct of the parent study and reviewed and commented on drafts of the manuscript. All authors read and approved the final manuscript.

## Pre-publication history

The pre-publication history for this paper can be accessed here:

http://www.biomedcentral.com/1472-6963/12/155/prepub
